# Reported thresholds of self-motion perception are influenced by testing paradigm

**DOI:** 10.1007/s00415-022-11032-y

**Published:** 2022-03-16

**Authors:** M. Pleshkov, N. Rondas, F. Lucieer, L. van Stiphout, M. Janssen, N. Guinand, A. Perez-Fornos, V. Demkin, V. van Rompaey, H. Kingma, R. van de Berg

**Affiliations:** 1 Division of Balance Disorders, Department of Otorhinolaryngology and Head and Neck Surgery, School for Mental Health and Neuroscience, Maastricht University Medical Center, Universiteitssingel 40, 6229 ET Maastricht, The Netherlands; 2 Faculty of Physics, Tomsk State University, Tomsk, Russia; 3 Faculty of Health, Medicine and Life Sciences, Maastricht University, Maastricht, The Netherlands; 4 School for Public Health and Primary Care (CAPHRI), Department of Methodology and Statistics, Maastricht University, Maastricht, The Netherlands; 5 Service of Otorhinolaryngology Head and Neck Surgery, Department of Clinical Neurosciences, Geneva University Hospitals, Geneva, Switzerland; 6 Department of Otorhinolaryngology and Head and Neck Surgery, Faculty of Medicine and Health Sciences, Antwerp University Hospital, University of Antwerp, Antwerp, Belgium

**Keywords:** Self-motion thresholds, Motion perception, Two-option paradigm, Twelve-option paradigm

## Abstract

**Background/objective:**

Different testing paradigms have been proposed to investigate perceptual self-motion thresholds. They can differ regarding the amount of possible motions that patients have to choose from. Objective of this study was to compare the two-option paradigm and twelve-option paradigm, to investigate whether reducing the choice options significantly influences the reported thresholds of self-motion perception of healthy subjects.

**Methods:**

Thirty-three volunteers with no prior vestibular complaints were included and sequentially tested with both paradigms at a random sequence. Perceptual self-motion thresholds were measured using a hydraulic motion platform in the absence of external visual and auditory cues. The platform delivered twelve different movements: six translations and six rotations. Each subject had to report the correct type and direction of movements. Thresholds were determined by a double confirmation of the lowest threshold, in combination with a double rejection of the one-step lower stimulus. Perceptual self-motion thresholds of both paradigms were compared using the mixed model analysis.

**Results:**

The twelve-option paradigm showed significantly higher reported thresholds for yaw rotations and translations left, right and down (*p* < 0.001), compared to the two-option paradigm. No statistical difference was found for rolls and translations up. No significant gender effect, learning effect and carry-over effect were present in any of the applied motion directions.

**Conclusion:**

Reported thresholds of self-motion perception of healthy subjects are influenced by the testing paradigm. The twelve-option paradigm showed significantly higher thresholds than the two-option paradigm. Results obtained with each testing paradigm should, therefore, be compared to paradigm-specific normative data.

**Supplementary Information:**

The online version contains supplementary material available at 10.1007/s00415-022-11032-y.

## Introduction

The vestibular organ consists of three semi-circular canals and two otolith organs. The semi-circular canals detect angular accelerations, while the otolith organs mainly detect linear accelerations and head tilt. Besides these motion cues from the vestibular organ, the brain also receives visual, somatosensory and auditory information and combines these inputs to maintain posture, gaze stabilisation and spatial orientation [1–3].

The clinically mostly used vestibular function tests investigate the vestibulo-ocular reflex and the vestibulo-collic reflex. There is not one standard diagnostic test for analysing the vestibular function, as the clinically applied tests are complementary to each other, and one test cannot replace another. All tests need to be executed and interpreted by a well trained professional [4, 5]. However, about one-third of the patients with complaints of dizziness and/or imbalance have normal vestibular test results. This suggests that either some vestibular disorders may not involve the vestibulo-ocular reflex, and/or the standard diagnostic tests available are not applicable to all vestibular complaints. Therefore, there seems to be a need for a clinical test that measures beyond vestibular reflexes [6].

A relatively new method for assessing (part of) the vestibular function is determining perceptual self-motion thresholds [7]. For this, the subject has to take place at a motion platform or sled that is able to move in different directions, with different accelerations. After every motion, it is checked whether the subject perceived the movement correctly. The perceptual thresholds can be determined by changing the acceleration and direction of the platform, according to the response of the subject. The threshold for each direction is determined by the lowest acceleration that can still be correctly perceived by the subject. Exclusion of the other somatosensory cues (vision, sense of hearing, sense of touch) is preferred, as they support the vestibular system in its spatial orientation. However, this is not totally possible, especially for somatosensory input [7, 8]. This, therefore, implies that mainly perceptual self-motion thresholds are tested, and not “pure” vestibular perceptual thresholds.

The advantage of testing perceptual self-motion thresholds could be that it does not depend on vestibular reflexes. Since different sensory mechanisms, other than reflex pathways, might be responsible for the perceptual responses [9–11], testing perceptual self-motion thresholds could be complementary to the other clinical tests [12]. Next to this, future development of a self-motion ‘vestibulogram’, might be a useful tool in the diagnostic work-up of vestibular disorders in clinic. This vestibulogram, adjusted to gender, age and current diseases, shows perceptual thresholds (in acceleration units) as a function of frequency, similar to the concept of an audiogram, where the auditory thresholds (in decibel units) are shown as a function of frequency [6, 8].

In literature, different methods are used to determine the perceptual self-motion thresholds. They mainly differ regarding (1) the type of platform or sled, (2) the type and amount of directions tested, (3) the stimulus profile, (4) testing time, and (5) paradigm for determining the thresholds. Regarding this latter, subjects can have either a two- or plural-option paradigm. In other words, subjects are or are not informed about the possible motion directions before each test. The amount of possible motions can be either two or more. The effect of cognition, knowing the amount of available options beforehand, may influence the sensitivity of the perceptual thresholds [8, 13–17]. Next to this, the chance of guessing the correct movement might increase with fewer choice options.

Objective of this study was, therefore, to compare two previously described paradigms [7, 15] for determining thresholds of self-motion perception (twelve-option versus two-option paradigm), to investigate whether reducing the choice options significantly influences the reported thresholds of healthy subjects, obtained with a more clinically oriented test [7].

## Methods

### Study design

A more clinically oriented test for self-motion perception was used. This was previously described [7], and will be discussed more in detail below. Regarding testing paradigm, two different paradigms for determining perceptual self-motion thresholds were tested: (1) twelve-option paradigm, and (2) two-option paradigm. Each subject underwent two trials: one with the twelve-option paradigm and one with the two-option paradigm. In between the trials, a short break was scheduled of about 15 min. Randomization was applied (using https://www.randomizer.org) across and within the paradigms, i.e., subjects were randomized into two nearly equal groups that started with either the twelve or the two-option paradigm, and the sequence of motion types provided in each paradigm was randomized as well. All tests were conducted by the same technician (NR). Perceptual thresholds were measured in acceleration units (m/s^2^ for translations, and deg/s^2^ for rotations).

### Setting

This study was conducted at Maastricht University Medical Center. Subjects were recruited at the university and hospital by addressing people personally and by distributing flyers.

### Subjects

Thirty-three healthy individuals (14 males and 19 females, age 22–72 years) participated in this study. To be included, they had to be able to climb three stairs to the platform and sit on the platform for at least 1 h and a half. Exclusion criteria comprised: vestibular and/or hearing complaints, headaches fitting the diagnostic criteria of migraine [18, 19], the use of antidepressants for anxiety or depression, and the use of other vestibulosuppressants, such as sleeping pills. All subjects completed a short questionnaire on beforehand, that was used to collect personal data (age and gender) and to screen for exclusion criteria.

### Perception platform

A hydraulic CAREN platform (Motek Medical BV, Amsterdam, The Netherlands) with D-flow 3.22.0 software was used for this study. The platform was programmed to move in twelve directions: six translations (up, down, left, right, forward, backward) and six rotations (yaw left, yaw right, roll left, roll right, pitch forward and pitch backward).

### Preparations

Subjects had to take place on a chair on the platform and were strapped with two seatbelts. To exclude visual cues, testing was performed in a dark room and subjects were blindfolded. A headphone was used to mask the sounds of the moving platform by playing earlier recorded platform sounds. This headphone was also used by the examiner to communicate with the subjects, to keep their attention during the test. Next to this, an infrared camera made it possible to observe the subjects during the test. The head of the subject was intentionally not fixed during the test, since the chosen study design aimed at mimicking a relatively natural situation of whole-body motion, in which only visual and auditory feedback were prevented as much as possible.

### Testing paradigms

#### Twelve-option paradigm

Seventeen subjects started with the twelve-option paradigm. Thresholds were determined during the same testing trial for all twelve motion directions possible (twelve-alternative). All subjects were informed about the total amount of motion types that were applied within this paradigm. The motion directions were randomly chosen by the examiner and started at the highest possible acceleration: 0.4 m/s^2^ for translations and 40 deg/s^2^ for rotations. After each motion the subject was asked to report both the type and direction of motion. In case of a correct answer, the stimulus was decreased with 0.03 m/s^2^ or 3 deg/s^2^. If the subject could not indicate the correct direction, the acceleration was increased by 0.03 m/s^2^ or 3 deg/s^2^.

#### Two-option paradigm

Sixteen subjects started with the two-option paradigm. This method was based on a two alternative choice paradigm. Before each motion subjects were informed about the type of motion including two options, for example “translation up or down” or “yaw left or right”. Therefore, subjects only had to report the direction of motion, instead of reporting the type of motion as well. Thresholds of four types of motions were determined: translations left and right, translations up and down, yaw left and right, and roll left and right [15]. All other elements of this paradigm were similar to the twelve-option paradigm.

#### Thresholds

Perceptual thresholds can be found by investigating a psychometric function, in which the relation between stimulus magnitude and level of correct answers are expressed. Thresholds are then often determined by finding the stimulus magnitude at which a performance level (e.g., 50% correct answers) is reached [20]. However, constructing the psychometric function was not the objective of this study. After all, investigating the psychometric function takes considerable time and this study involved a more clinically oriented test. It was, therefore, chosen to determine perceptual self-motion thresholds by a double confirmation of the lowest threshold in combination with an double incorrect response at the acceleration one step below the threshold [7]. Furthermore, this study aimed at determining the reported self-motion perception thresholds as an “end result” of all variables involved (including sensitivity of the subject, contribution of the somatosensory system, amount of choice options etc.). Taking all these factors into account, the reported thresholds reflected the ability of self-motion perception as a result of all variables involved, not the vestibular perceptual threshold as a psychometric parameter.

### Stimulus profile

**Fig. 1:**
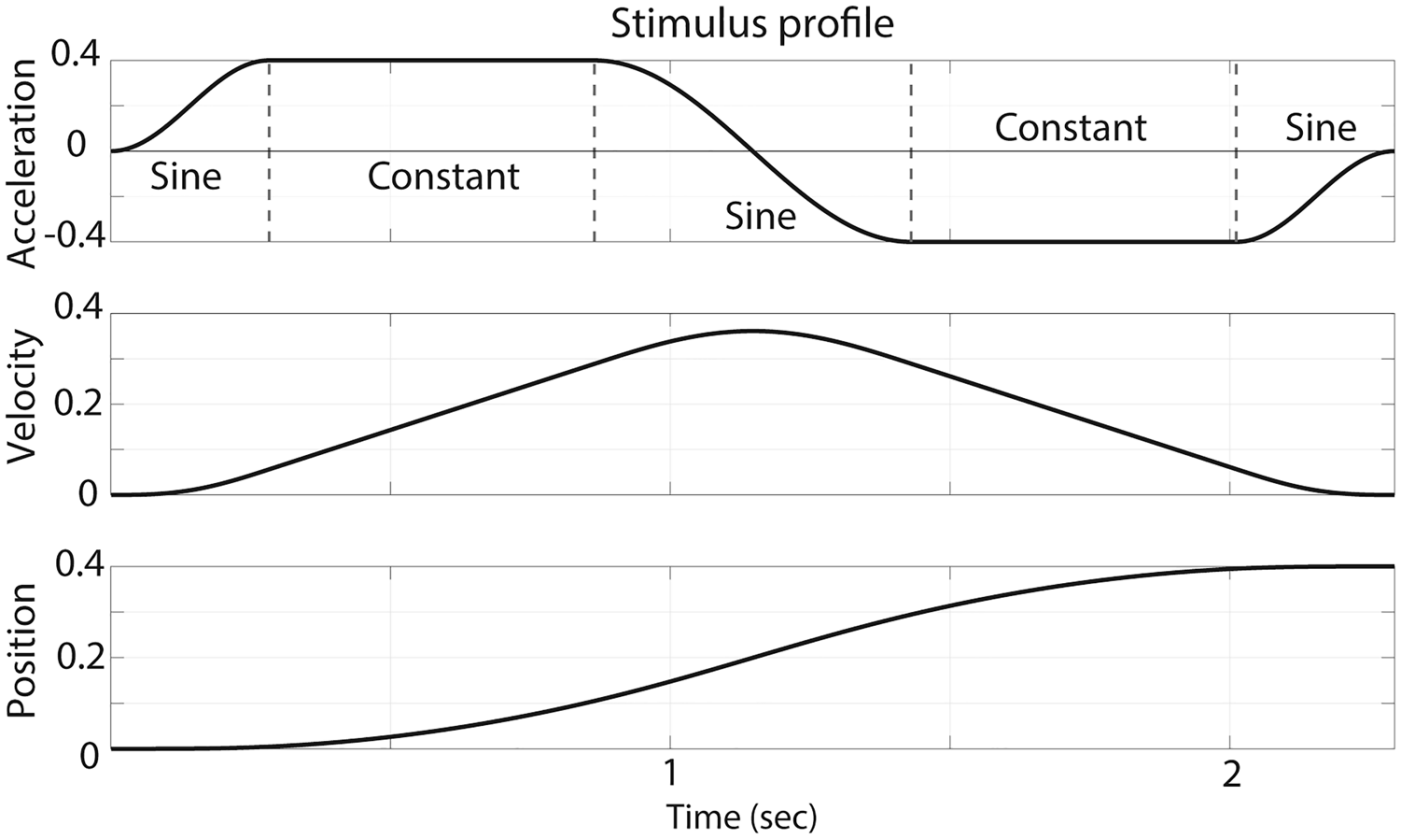
Motion profile used. The stimulus profile was composed of subsequent sine and constant functions. The units of measurement were either (m/s^2^, m/s, m) or (deg/s^2^, deg/s, deg) depending on the movement type: translational or rotational, respectively

The applied motion stimulus profile was previously described [7]. For short, the platform motion profile was developed to provide the desired linear or rotational acceleration as long as possible. Every motion comprised an acceleration and deceleration of equal duration. The rise and decay of the acceleration followed a sinusoidal profile, to smoothly reach the selected magnitude (Fig. 1). Due to platform limitations, frequency and duration of stimulus, but not displacement amplitude, varied for each separate motion stimulus. In between tested movements, the platform moved into its new position using subthreshold movements.

### Data analysis

R v.3.5.2 was used to compare both paradigms. A linear mixed model analysis was performed for each motion direction to investigate gender effect, learning effect, paradigm effect and carry-over effect. The learning effect was evaluated to check whether repeated measurements (first trial versus second trial) influenced the perceptual self-motion thresholds, independent of the paradigm used. The paradigm effect was investigated to check any influence of the tested paradigm (twelve-option versus two-option) on the perceptual self-motion thresholds. The carry-over effect was related to the influence of the variable “trial” on “paradigm” regarding the perceptual self-motion thresholds. Therefore, gender, trial number (first or second), paradigm (twelve- or two-option), and trial-paradigm interaction were applied as fixed factors, while the participant number was applied as a random factor. The threshold per motion direction was considered the dependent variable in the mixed model. Following the top-down procedure, the trial-paradigm interaction was tested first, followed by the factors gender and trial. They were not significant and, therefore, excluded from the mixed model. Since translations forwards–backwards and pitch forwards–backwards were not tested in the two-option paradigm (as in previous literature [15]), and thresholds of rolls could not reliably be determined due to physical limitations of the platform (see “Results”), outcomes of six directions were compared using the mixed model. These motions involved translations left and right, translations up and down, and yaw left and right. The significance of fixed effects was investigated using Type III tests ANOVA. The Bonferroni correction (*n* = 6) for multiple comparisons was applied to the significance level *alpha* = *0.05*.

## Results

**Fig. 2:**
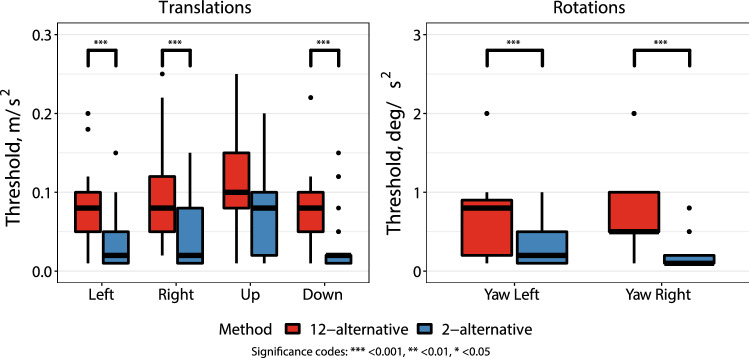
Reported thresholds of self-motion perception of translations (**A**) and rotations (**B**), tested with the twelve option-paradigm (red) and two-option paradigm (blue). Boxes represent the interquartile ranges, bold black horizontal lines the medians, and upper and lower whiskers the extreme lines. Outliers are represented by black dots

All measured perceptual self-motion thresholds are shown in Table 1 as mean ± SD. Figure 2 presents the comparison of perceptual self-motion thresholds between the twelve-option paradigm and the two-option paradigm. The twelve-option paradigm showed significantly higher perceptual self-motion thresholds than the two-option paradigm (*p* < 0.001), except for translations up, and rolls left and right. Regarding translations up, thresholds of the twelve-option paradigm were higher, but not significant. During rolls left and right, the lowest measurable thresholds (0.1 deg/s^2^) were obtained in nearly all subjects. Due to physical limitations of the platform, lower accelerations in these planes could not be provided for these types of movements, and therefore, these thresholds were considered as “not determined”. Reported thresholds of self-motion perception did not differ significantly between male and female subjects and no significant learning effect and carry-over effect were present in any of the applied motion directions.

**Table 1:** Mean reported thresholds of self-motion perception (± SD) presented for translations and rotations for both tested paradigms

	Paradigm		Units
	12-option	2-option	
Translation Left	0,08 (0,05)	0,04 (0,04)	m/s^2^
Translation Right	0,10 (0,06)	0,04 (0,04)	
Translation Up	0,14 (0,10)	0,11 (0,14)	
Translation Down	0,11 (0,11)	0,03 (0,04)	
Translation Forward	0,11 (0,06)	–	
Translation Backward	0,11 (0,07)	–	
Yaw Left	1,51 (1,75)	0,34 (0,30)	deg/s^2^
Yaw Right	1,04 (1,20)	0,24 (0,24)	
Roll Left	ND	ND	
Roll Right	ND	ND	
Pitch Forward	0,31 (0,86)	–	
Pitch Backward	0,21 (0,36)	–	

*ND* the threshold value could not be determined

## Discussion

This study investigated the reported self-motion perception thresholds of healthy volunteers, when using two different testing paradigms: the two-option and twelve-option paradigm. The reported self-motion perception thresholds were measured with a more clinically oriented test, in which only visual and auditory feedback were prevented as much as possible, and in which thresholds were determined as a double confirmation of the lowest threshold in combination with an double incorrect response at the acceleration one step below the threshold. This implies that the obtained thresholds reflected the “end result” of many contributing factors (e.g., vestibular system, somatosensory system, etc.), and not the “pure vestibular threshold” as a psychophysical parameter.

The obtained values of the reported thresholds were compared among testing paradigms. The study showed that reported thresholds of healthy subjects are influenced by the testing paradigm. After all, reported thresholds of the twelve-option paradigm were (mostly) significantly higher than those determined by the two-option choice paradigm. No carry-over nor learning effect was present in any of the applied motion directions. This implies that results obtained with each testing paradigm should, therefore, be compared to paradigm-specific normative data.

The difference in reported thresholds might be explained by two factors. First, the chance of guessing the correction motion direction was higher in the two-option paradigm than in the twelve-option paradigm. This could have decreased the thresholds in the two-option paradigm. No correction for guessing was applied in this study, since the amount of tested stimuli depended on the amount of correct answers, and, therefore, varied highly between subjects. Second, the pre-knowledge of the type of motion in the two-option paradigm could have positively influenced the subjects perception of motion (i.e., the subject was specifically focussed on a certain motion plane during the test). This might have also lowered the thresholds in the two-option paradigm. However, whether the sensitivity for motion stimuli of the healthy subjects truly differed between testing paradigms, can only be determined by constructing a psychometric function for both testing paradigms (see “Methods”) [21]. However, this was beyond the scope of this article. This study showed that paradigm-specific normative data should be used to interpret results obtained within a subject.

The roll left and roll right threshold values could not be determined due to physical limitations of the motion platform: the platform could not provide lower accelerations than already perceived by the subjects. These low magnitudes of roll thresholds could be explained by the fact that the somatosensory system might play a more prominent role in rolls, than in the other motions [7].

Regarding translations up, it was noted during testing that many subjects had difficulty in recognizing upward motions. This was reflected in the highest overall mean of perceptual self-motion thresholds. Whether this might have influenced the non-significant difference between testing paradigms, cannot be determined with certainty.

### Implications for future research

Type of testing paradigm influences the reported thresholds of self-motion perception. Studies regarding this matter should, therefore, not be compared to each other, without taking these differences into account. Next to this, performance was variable between subjects. An association between increasing reported thresholds and age was already described using this clinically oriented test and a more research oriented test [7, 15]. This variability might lead to the question whether the vestibular organ is really that direction sensitive for perception, or whether it mainly has a signaling function, whereas other sensory systems (e.g., visual, propriocepsis) are mainly used to determine the direction of motion. After all, divers under water in dark and subjects buried alive in an avalanche, have difficulties orienting themselves although gravity is still detected by the vestibular organs [22]. This signaling function at least implies that there will probably be another perceptual self-motion threshold: the threshold of perceiving motion itself, without being able to detect the right direction of motion. This might be explored in future research.

### Implications for clinic

The more clinically oriented approach used in this paper, can pave the way for a relatively simple and faster way to assess perceptual self-motion thresholds in clinic, using normative data. Within this context, it should be noted that the twelve-option paradigm takes about 45–60 min, while the two-option paradigm takes about 30–45 min. This could be an important detail for clinical practice.

The motion platform used in this study is relatively expensive. This might hinder implementation in daily routine clinical practice. It could, therefore, be considered to use a less-expensive motorized rotatory chair [23]. A rotatory chair might be able to provide a broader range of velocities, but it provides less movements: only yaw rotations. However, the clinical value of testing additional movement types is not yet fully determined.

### Limitations of the study

Although all subjects wore headphones with a masking background sound, some subjects could still hear the sounds of the platform during the tests at high accelerations [7]. However, the sounds did not provide any information about motion direction and were present only in the range of accelerations that were much higher than threshold values. Therefore, these audible sounds could not have influenced the measured outcome. Second, some subjects below 50 years could still feel the subthreshold movements of the platform that were used to get the platform into a new testing position. For future testing, parameters of these subthreshold movements can be lowered to such an extent that subjects will less likely to be able to feel them. Third, vestibular reflexes were not evaluated in the study population. Subjects were selected based on the absence of the vestibular complaints and vestibular deficits. This most likely would not have hindered the objective of the study, since patients served as their own controls.

## Conclusion

Reported thresholds of self-motion perception of healthy subjects are influenced by the testing paradigm. The twelve-option paradigm showed significantly higher reported thresholds than the two-option paradigm. Results obtained with each testing paradigm should, therefore, be compared to paradigm-specific normative data.

## Supplementary Information

Below is the link to the electronic supplementary material.

Supplementary file1 (MP4 4264 KB)
